# Tunable Drug Release Rate Using Modular Oral Dosage Forms

**DOI:** 10.3390/pharmaceutics15071905

**Published:** 2023-07-08

**Authors:** Mario A. Cano-Vega, Laura M. Arango-Salazar, Rodolfo Pinal

**Affiliations:** 1 Department of Agricultural and Biological Engineering, Purdue University, West Lafayette, IN 47907, USA; mcanoveg@amgen.com; 2 Department of Industrial and Physical Pharmacy, Purdue University, West Lafayette, IN 47907, USA

**Keywords:** solubility, pharmaceutical films, drug release kinetics, controlled release, modular dosage forms, spring-parachute, multi-layer, additive manufacturing

## Abstract

Oral dosage forms with adjustable drug release profiles were prepared using progesterone (PGR) as a poorly-soluble model drug. The dosage forms were made as stack assemblies of functional modules. The modules were made as PGR-carrying HPMC films cut into wafer-like circular pieces. Two types of modules were used in the study; one exhibited comparatively fast drug release and the other slow release. The fast vs. slow release of each type of film utilized resulted from the grade of HPMC used in each case. Drug loading in the assembly was controlled through the total number of modules. By adjusting the proportions of the two types of modules, it is possible to fine-tune the drug release rate of the multi-layer assemblies to a wide range of profiles, bracketed between a high and low end, corresponding to the inherently fastest or slowest release obtainable with the specific materials and procedures employed. This procedure is suitable for adjusting the spring-and-parachute parameters for enhancing/optimizing the bioavailability of poorly-soluble drugs, and for developing patient-centric formulations.

## 1. Introduction

The poor aqueous solubility of a large proportion of drugs is a prevalent challenge in pharmaceutical development. About 40% of new drugs exhibit poor aqueous solubility [1]. Since drug absorption requires the drug to be dissolved in a predominantly aqueous medium, poor drug solubility is closely connected with low bioavailability. The search for techniques for increasing the solubility and/or accelerating the dissolution rate of drugs is a significant thrust in drug development. Salt formation is arguably the most widely used approach for increasing the solubility/dissolution rate of poorly-soluble drugs. In fact, the majority of drugs approved in the US are formulated in the form of salts [2]. In simple terms, dissolution of a salt in the GI tract results in the non-equilibrium condition of supersaturation relative to the solubility limit of the free acid or free base species (intrinsic solubility) of the drug. Even though any supersaturation condition generated by the dissolution of a salt is only transient, it nevertheless provides a time window of dissolution at substantially higher concentrations than the equilibrium solubility limit. The supersaturation phenomenon has been successfully exploited beyond salts and short-lived supersaturation conditions. Specifically, the spring-and-parachute effect [3] has been amply exploited with different systems, including amorphous [4] and cocrystal [5] dispersions. Accordingly, it is possible to enhance and/or optimize the bioavailability of poorly soluble drugs by utilizing formulation approaches that prolong the time duration of supersaturation conditions. The potential of the spring-and-parachute to enhance the bioavailability of orally administered drugs is expected to apply, whether the poorly-soluble drug falls into category II (high permeability) or IV (low permeability) of the BCS classification system [6]. The aim of this report is to present a modular formulation design approach for orally administered poorly-soluble drugs. The oral dosage forms produced with this approach have tunable drug release kinetics, a feature that can be exploited for enhancing/optimizing drug absorption and ensuing bioavailability by generating drug supersaturation, prolonging the duration of supersaturation conditions, or both.

## 2. Rationale

The underlying concept of the formulation design presented here is that the oral dosage form is conceived as an assembly of prefabricated functional modules. Each module is a thin wafer, and the dosage form is a stack of such modules. The wafers are pieces taken from a pharmaceutical film, cut into a circular shape for use in an oral capsule. The use of capsules as carriers, as used in this report, is not essential; the wafers can also be made into a 3D stack to create a multi-layer tablet-like assembly [7]. Pharmaceutical films have been used as drug delivery systems for nearly fifty years [8]. However, it was in the 1990s and early 2000s when films became an important element of the pharmaceutical dosage form repertoire [9,10,11]. Moreover, it was in 2010 when the FDA approved the first prescription pharmaceutical film product (Zuplenz^®^, ondasentron) [12]. More recently, significant progress has been made in pharmaceutical film technology [13,14,15]. We should emphasize that the dosage form of the approach presented here is not a thin film meant to be placed in the buccal cavity. Instead, the dosage form is a traditional capsule (or a solid body with the dimensions of an oral tablet) meant to be swallowed as such. However, the approach used here exploits the features and advantages offered by pharmaceutical films, by utilizing them as modules in modular dosage forms.

**Figure 1:**
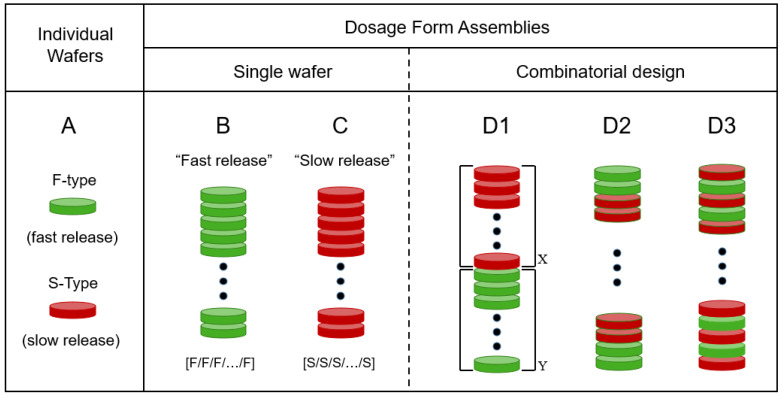
Depiction of the modular dosage forms of this study. (**A**). Individual film wafers of two types, F and S, denoting fast and slow drug release, respectively. (**B**). All-F-wafer dosage form, made exclusively with fast-release wafers. (**C**). All-S-wafer dosage form, made exclusively with slow-release wafers. (**D**). Possible combinations with two types of wafers (S and F) in inventory. The D1, D2 and D3 configurations depict different possible arrangements attainable with the same number of green (F) and red (S) wafers. Solid dots represent an arbitrary number of repetitions of the pattern shown at either end of the depiction. The D1 configuration was chosen for this study (see text), X and Y denote the number of S and F wafers, respectively in the stack.

Figure 1 presents a depiction of the modular approach of this report. The basic unit is a wafer cut from a pharmaceutical film. In the type of system reported here, there are only two types of wafers. One wafer type, denoted as F, exhibits fast drug release. The other wafer type, denoted as S, shows (comparatively) slow drug release. We should point out that the “fast” (F) and “slow” (S) designations used here are relative to each other; one type of film (F) releases the drug faster than the other (S), as discussed below. The oral dosage form assembly is made by stacking the desired number of film wafers. Accordingly, if all wafers are of type F (all-F-wafer assembly), the resulting dosage form, as a whole, will exhibit a comparatively fast drug release. Conversely, if all wafers in the stack are of type S (all-S-wafer stack), the dosage form will show a slow drug release, relative to the all-F-wafer stack. However, this study will show that the modular approach makes it possible to produce oral dosage forms whose drug release rate can be adjusted as needed, in a predictable manner, by combining F and S modules in the stack assembly. Three possible configurations for tunable release kinetics are depicted under the D1–D3 representations in Figure 1. The combinatorial nature of the type of modular dosage form of this study makes the number of possible configurations quite large. This aspect is viewed as advantageous because of the flexibility it offers in terms of dosage form design and ensuing drug release profile. However, the aim of the present study is to utilize the simplest possible configuration (D1 in Figure 1) as proof of concept, to show adjustable drug release kinetics using this modular approach.

## 3. Materials and Methods

Progesterone (PGR) was used as a model drug of a poorly-soluble drug in this study. The aqueous solubility of PGR has a value of 0.01 mg/mL [16]. Progesterone is used in hormone replacement therapy for women, and there are essential reasons for having tunable drug release capabilities in this particular type of therapy. First, in hormone replacement treatment, patient-centric formulations are highly desirable; different patients respond noticeably differently to the same drug regimen. So, optimal therapy requires the ability to adjust the dose and systemic exposure according to the needs voiced and assessed by the patient and physician, respectively. Second, even for the same hormone replacement patient, therapy needs are not locked in, as they change over time. Consequently, there is a continued need for adjusting the therapy regimen as needed. Third, PGR absorption in the GI tract is partially subject to active transport [17]. Active transport is subject to saturation [18], such that formulation approaches that only increase the concentration of the dissolved drug (spring) may not always be the best approach for optimizing systemic drug exposure. The ability to extend the parachute effect is also a crucial aspect of optimizing drug release and subsequent absorption.

### 3.1. Solvent Casting of PGR-Loaded Fast-Release (F) Wafers

Polymer films were produced using the solvent casting technique. Two formulations with different release rates, fast and slow. Studies reported in the literature indicate that increasing the HPMC viscosity through the use of different grades, leads to decreasing release rates [19]. HPMC 2910-5 MPa (low viscosity) and HPMC 2906–4000 MPa (high viscosity) were selected in this study due to their differences in viscosity. In vitro release studies performed on tablets coated with each of these polymers showed noticeably different release kinetics [20]. 

First, the composition of the films in this study was optimized, varying the concentration of HPMC as well as the volume of the cast solution. In order to evaluate the suitability of the HPMC solutions for the solvent casting process, the different film-forming solutions were cast onto a polyvinyl chloride (PVC) substrate surface using an adjustable film applicator (doctor blade). The films were peeled off from the casting substrate, and the physical appearance and thickness were evaluated. Then, the drug load was optimized by varying the amount of drug added to the optimized HPMC solution. Several suspensions were prepared with different drug contents. The film-forming suspensions were cast onto a PVC substrate, and the drug content was quantified using a spectrophotometric method. The target drug content was established at 1 mg of PGR per 8 mm diameter PGR-carrying wafer. The fast-release (F) formulation was prepared as follows. Briefly, film-forming dispersions of PGR were prepared by dissolving 3.10 g of glycerin (plasticizer) in 50 mL of deionized water and heating to 80 °C. Then, 0.02 g of surfactant, SDS (sodium dodecyl sulfate), was added and stirred until fully dissolved. Subsequently, 2.50 g of PGR was homogeneously dispersed into the solution. Finally, 6.25 g of HPMC 2910-5 MPa (Methocel E5 Premium LV, Dow Chemical, Midland, MI, USA) was added and homogeneously dispersed into the aqueous dispersion while still warm. The resulting dispersion was cooled down to room temperature and stirred overnight to allow complete HPMC dissolution/distension. The final film-forming suspension was cast onto a PVC substrate surface using a doctor blade. The films were peeled off from the casting substrate and stored in a polyester bag at room temperature for further analysis. The dry films were cut into circular (fast-release) F-wafers with a diameter of either 8 or 15 mm, using a punch-cut tool. The thickness of the wafers was measured using a digital micrometer (Mitutoyo, Kawasaki, Japan). The thickness of the F-wafers was 0.079 ± 0.004 mm.

The slow-release (S) formulation was prepared by dissolving 1.2 g of glycerin (plasticizer) in 50 mL of deionized water and heating to 80 °C. Then, 0.02 g of SDS was added and stirred until fully dissolved. Subsequently, 0.8 g of PGR was homogeneously dispersed into the solution. Finally, 1.5 g of HPMC 2906–4000 MPa (Methocel K4M Premium CR, Dow Chemical, Midland, MI, USA) was added and homogeneously dispersed into the dispersion while still warm. The resulting dispersion was cooled down to room temperature and stirred overnight to allow complete HPMC dissolution/distension. The final film-forming suspension was cast onto a PVC substrate surface using a doctor blade. The films were peeled off from the casting substrate and stored in a polyester bag at room temperature for further analysis. The dry films were cut into circular (slow-release) S-wafers with a diameter of either 8 or 15 mm, using a punch-cut tool. The thickness of the wafers was measured using a digital micrometer (Mitutoyo, Kawasaki, Japan). The thickness of the S-wafers was 0.077 ± 0.008 mm.

### 3.2. Preparation of 3D Assemblies

The drug content of the PGR F-films was 20% (*w*/*w*), corresponding to 1 mg of PGR per PGR wafer with 8 mm diameter. Similarly, the drug content of the PGR S-films was 22% (*w*/*w*), corresponding to 1.1 mg of PGR per PGR wafer with 8 mm diameter. The dose control capability was tested by stacking the required number of PGR wafers (8 mm diameter) to give 15, 30, or 50 mg of total PGR content in the corresponding dosage form stack. For notation purposes, an all-F-wafer dosage form assembly, that is, one made exclusively of fast-release, F-wafers, is denoted in the following fashion: [F/F/F…/F] and depicted in Figure 1B. Similarly, an all-S-wafer dosage form assembly, i.e., one made exclusively of slow-release, S-wafers, is denoted in the following fashion: [S/S/S…/S] and depicted in Figure 1C. Pharmaceutical films have been traditionally utilized, for the most part, because of the fast drug release possibilities they offer [8,10,21,22]. More recently however, it has been pointed out that pharmaceutical films exhibiting prolonged drug release offer valuable complementary advantages [23,24]. The type of assemblies depicted in Figure 1, depictions B and C, correspond to the fast and slow drug release rates, respectively, achievable with the specific materials and procedures employed to make the films in the example presented here.

Each wafer (layer) in a dosage form assembly represents a functional module. The ability to control the drug release rate utilizing this type of modular dosage form design was tested by combining two types of wafers. Specifically, as separate stacks made of either F- or S-type wafers and put together into a single dosage form. These new dosage forms are graphically represented in Figure 1, depiction D1, namely {[F/F/F…/F]*_X_*–[S/S/S…/S]*_Y_*}, where *X* and *Y* denote the number of F- and S-wafers in the stack, respectively. Accordingly, the total number of wafers (*X* + *Y*) used to make the dosage form directly controls the total drug dose. For *X* = 0 and *Y* = 0, the dosage form will exhibit the slowest and fastest drug release rate, respectively, obtainable with the inventory of S- and F-modules used to make the assemblies in this example. The number of possible configurations obtainable with only two module types (e.g., F- and S-wafers) is indeed vast, and the effect that the combinatorial design has on drug release is worthy of further investigation. Three possible 2-wafer-type assembly configurations are depicted in Figure 1D. In this report however, we limit ourselves to the most straightforward configuration, as illustrated in Figure 1, depiction D1. The stack assemblies were placed into gelatin capsules as the final oral dosage form. The final dosage form in this approach does not have to be a capsule. In fact, assemblies of this type resembling traditional tablets have been reported [7]. However, patients undergoing treatment with PGR typically receive their medication in the form of capsules. The procedure described above was utilized to produce five different modular dosage forms, each with a different F:S wafer ratio: 100:0, 73:27, 50:50, F 27:73, and 0:100. The same basic procedure can be used to produce dosage forms with a different active pharmaceutical ingredient (API). Different API strength is achievable by adjusting the total number of API-carrying wafers, while different drug release kinetics are achieved by varying the relative proportions of F (fast-) and S (slow-) release wafers. Namely, dosage forms are made with a discrete number of modules (wafers), each carrying a precise amount of drug. The total number (F + S) of wafers establishes the drug loading in the dosage form, while the F:S ratio determines the drug release kinetics from the dosage form.

### 3.3. Drug Release from Individual Wafers and Wafer Assemblies

Methods for evaluating drug release from pharmaceutical films have been reported in the literature [25]. In this study, the in vitro drug release kinetics from individual F- and S-wafers and multi-layer assemblies were characterized using the rotating basket method (Apparatus I, DISTEK, dissolution system, North Brunswick, NJ, USA).

Individual F- and S-wafers with a diameter of 15 mm were characterized independently to assess the release kinetics of the individual wafer formulations. Each individual F- or S-wafer was placed at the bottom of a dissolution basket. The baskets containing the individual films were submerged in 300 mL of release medium. The volume of the release medium was chosen to maintain the PGR concentration in the dissolution medium above the limit of quantification and within the limits of a previously established calibration curve.

Alternatively, the dissolution studies for the full modular dosage form assemblies were designed to demonstrate the feasibility of the modular dosage form to control the drug release kinetics. For this purpose, each stack assembly was carefully placed into a hard gelatin capsule. The capsule was placed at the bottom of a dissolution basket. The baskets were submerged in 900 mL of release medium. The temperature of the water bath was maintained at 37 ± 1 °C during testing. The characterization of the release kinetics was monitored for 300 min, irrespective of the fraction of undissolved drug. Progesterone is a very poorly soluble drug. Accordingly, the 30 mg drug loading corresponds to a concentration in the dissolution medium in excess of 3300-fold the aqueous solubility of the drug. Therefore, dissolution testing of PGR requires a medium suitable for such a combination of drug amount and drug solubility. The release medium was 0.1 N HCl/ethanol (85:15% *v*/*v*). Ethanol was added to the dissolution medium as a co-solvent to help with the solubilization of the PGR. The composition of the dissolution medium was not intended to mimic the physiological conditions of the GI tract. The medium was specifically optimized for this study, serving characterization purposes and allowing objective comparisons among the different F:S ratios used to make the modular assemblies. Sampling with volume replacement (10 mL) was carried out at predetermined time points, specifically, 15, 30, 45, 60, 90, 120, 180, 240, and 300 min for modular dosage forms, 10, 20, 30, 40, 50, 60, 75, 90, and 120 min for individual S-wafers, and 2, 4, 6, 8, 10, 15, 30, 45, and 60 min for individual F-wafers. Sampling time points were selected so that the PGR concentration in the dissolution medium was greater than the limit of quantification (especially for the individual wafers), thus allowing characterization of the shape of the release profile. All samples were filtered through 0.45 µm membranes and diluted as needed for analysis with fresh release medium. Absorbance-based assays were carried out using a Varian Cary 300 Bio (Palo Alto, CA, USA) spectrophotometer at a wavelength of 249 nm. The concentration of PGR was determined using a previously generated calibration curve. The release profiles were constructed by plotting the percent of PGR released (%F) as a function of time. Measurements were performed in triplicate, and the average values were reported for individual wafers or multi-layer assemblies.

The uniformity of the individual F- and S-wafers was verified as follows: ten circular samples with a diameter of 8 mm were punch-cut from the film at random locations. Each piece was dissolved in 50 mL of 0.1 N HCl/ethanol (85:15% *v*/*v*) and stirred for 2 h using a magnetic stirrer. The obtained solution was diluted as needed with fresh 0.1 N HCl/ethanol solution for UV-Vis spectrophotometric analysis. Absorbance was measured at 249 nm, and the concentration was calculated using a calibration curve. From the assay, the average PGR content in the F-units was 103.3% ± 4% and 100.5 ± 1% in the S-units.

## 4. Results

### 4.1. Drug Release from Individual Fast- (F) and Slow- (S) Release Modules

**Figure 2:**
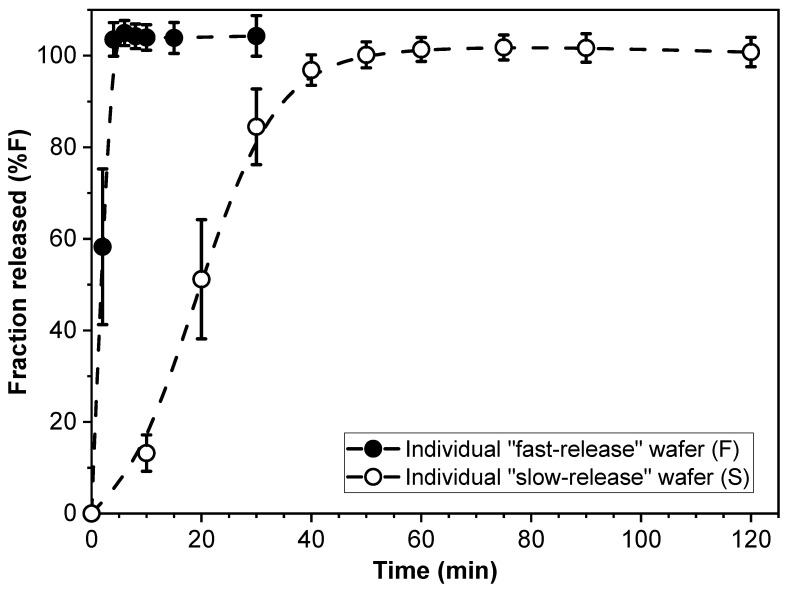
Cumulative percent release (%F) of PGR from individual “fast-release” (F-) and “slow-release” (S-) wafers.

The drug release kinetics of individual F- and S-wafers (15 mm diameter) were characterized, and the results are shown in Figure 2. As expected, drug release was substantially faster from the F-wafers, with essentially 100% of the drug load released within approximately 5 min. In contrast, completion of PGR release took about 60 min for the individual S-wafers.

The profiles in Figure 2 support the notion that the modular multilayer stack configuration can make dosage form assemblies of PGR that exhibit different drug release rates. Before exploring the aspect of tunable controlled release however, we investigate the drug release performance of the simplest type of modular dosage form, i.e., dosage forms made entirely with a single type of functional wafer.

### 4.2. Total Drug Content in the Dosage Form

**Figure 3:**
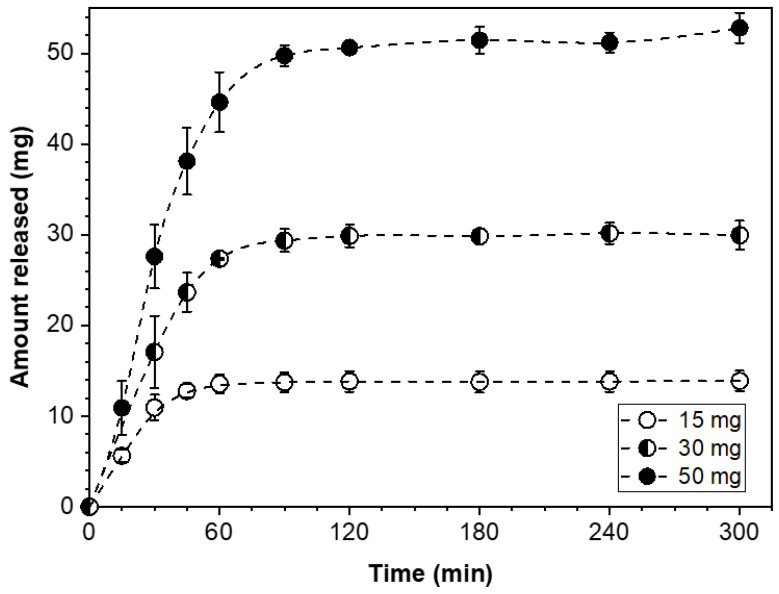
Cumulative amount of PGR released from all-F-wafer dosage form assemblies, i.e., [F/F/F…/F] configuration, with different dose strengths.

Precise dose control in the modular dosage forms of this study was accomplished by taking the number of PGR-bearing wafers (F- or S-type) needed to build the corresponding dosage form assembly carrying the desired drug loading. Accordingly, the dose customization of a modular solid dosage form can be readily adjusted by incorporating the necessary number of API-carrying wafers to confer the desired drug content to the dosage form. Modular dosage form assemblies with drug loadings of 15, 30, or 50 mg and configuration [F/F/F…/F], that is, an all-F-wafer type, were prepared. The drug release profiles for these dosage form assemblies are shown in Figure 3.

The drug release profiles in Figure 3 show that the plateau values at the 15, 30, and 50 mg mark coincide with the drug content of the corresponding dosage form assembly. These results show that the drug release is by all accounts complete within 60 to 120 min, depending on the dose level for modular all-F-wafer dosage forms, i.e., made exclusively of PGR-bearing F-type film wafers.

### 4.3. Dosage Forms with Fast and Slow Drug Release

**Figure 4:**
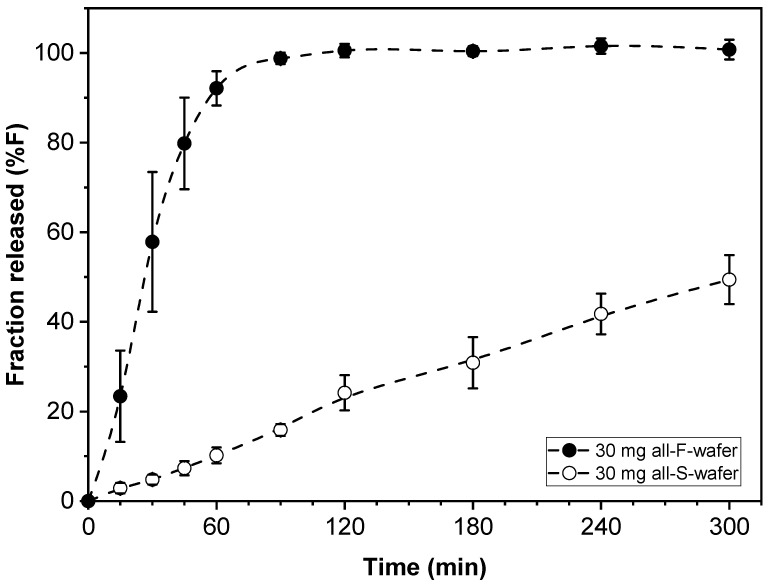
Drug release profiles of dosage forms made with 100% F-wafers (all-F-wafer) and 100% S-wafers (all-S-wafer), exhibiting comparatively “fast” and “slow” drug release, respectively. All dosage forms contain 30 mg of PGR.

Dosage form assemblies of all-F-wafer or all-S-wafer configuration were prepared to assess the range of drug release rates obtainable with the materials and procedures used in this study. That is, each type of wafer used in this approach exhibited inherent drug release characteristics. Such characteristics depend on the properties of the specific types of API and excipients used, the process utilized to make the films, and other attributes, such as the thickness of the film and geometry of the wafers. Dosage form assemblies with 30 mg drug loading made exclusively from either F- (all-F-wafer) or S- (all-S-wafer) wafers were prepared, and their corresponding drug release profiles assessed. The drug release profiles from the all-F-wafer and all-S-wafer dosage forms are shown in Figure 4. With everything else equal, the drug release profiles in Figure 4 are expected to provide the high and low end of the range of adjustable drug release rates obtainable by combining F- and S-type wafers to make the dosage form.

### 4.4. Customization of Drug Release Profile

**Figure 5:**
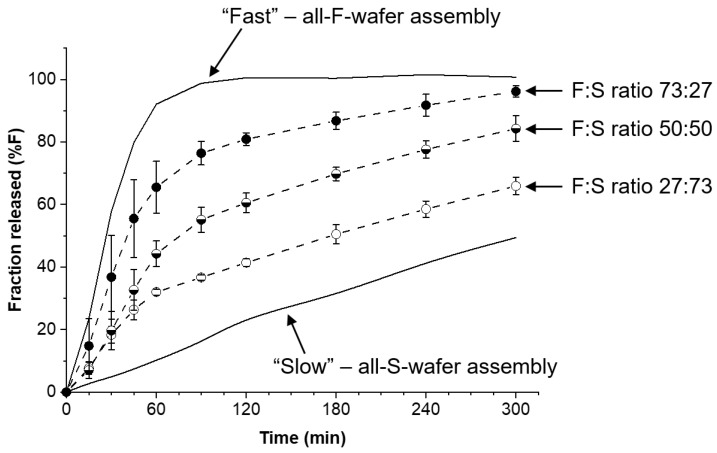
Adjustable drug release rate from modular dosage forms. Effect of proportion of individual fast- (F) and slow- (S) release wafer modules on the drug release of the final assembly. Assemblies were prepared according to the D1 configuration in Figure 1. Solid lines represent the high and low end of the drug release rate for the assembly under the conditions utilized in this example.

Modular dosage forms containing 30 mg PGR were prepared using different proportions of F- and S-type wafers, and the drug release rate for each configuration was assessed. Figure 5 shows the drug release profiles obtained from dosage forms containing F:S wafer ratios of 73:27, 50:50, and 27:73. In principle, precise 75:25 and 25:75 ratios can be used, but in the example presented here, the experimentally determined drug content of each type of wafer was taken into account to achieve the desired 30 mg total PGR in the dosage form. The solid lines in the plot represent the bracket for high and low drug release rates (see Figure 4) obtainable with the materials and procedures utilized to make the films and wafers used in this study. The high and low end profiles are labeled as “Fast” and “Slow” in Figure 5, respectively. The drug release profiles in Figure 5 show that with no more than two types of pharmaceutical films, wafers can be cut to make dosage forms with adjustable drug release rates. The percentage of drug released at 120 min was arbitrarily selected for comparison. The release profiles show that nearly 100% of the PGR was released after 120 min for the all-F-wafer dosage form. In contrast, only 20% of the PGR was released at the same time point for the all-S-wafer dosage form. Depending on the F:S wafer ratio, intermediate extents of drug release for the same time point can be achieved as desired, e.g., 40%, 60%, and 80% drug release for the ~1:3, 1:1, and ~3:1 F:S ratios, respectively.

### 4.5. Model for Drug Release Kinetics

It has been reported that the type of modular assembly used in this study behaves as a single-body system whose release kinetics are dictated by the initial geometry of the assembly. Moreover, the release kinetics of the assemblies can be successfully fitted to a conventional model developed for traditional dosage forms [7]. This attribute was exploited to model the release kinetics from assemblies containing combinations of S- and F-wafers, based solely on the release kinetics from all-S-wafer and all-F-wafer assemblies.

In the kinetic model presented below, the total amount of drug released at the time *t* from the whole dosage form assembly {[F/F/F…/F]*_X_*–[S/S/S… /S]*_Y_*} is considered as the sum of the partial contributions from the all-F-wafer and the all-S-wafer sections, [F/F/F…/F]*_X_* and [S/S/S…/S]*_Y_*, respectively, in the assembly (*X* and *Y* denote the number of F- and S-wafers in the stack, respectively, see Figure 1D). The first step is to fit the drug release data from the all-F-wafer and all-S-wafer assemblies to elucidate the kinetic model that fits the drug release data in each case.

The drug release profile is adequately described by a first-order expression for assemblies of the all-F-wafer type. Accordingly:

(1) $$F_{D}^{F}=100\;[1-\exp(-k_{1}\;t)]$$

where $F_{D}^{F}$ is the percentage of drug released at time *t* and *k*_1_ is the first-order kinetic constant.

It is important to point out that the release kinetics of the all-S-wafer assemblies is not expected to follow a zero-order pattern. However, during the timeframe of this study, the drug release from the all-S-wafer assemblies exhibited characteristics resembling zero-order release. Therefore, for purposes of model fitting in this study, the drug release from the all-S-wafer assembly is treated as a “zero” order profile. This practical aspect of the model is of value for application purposes. In principle, any empirical fit (e.g., a polynomial, a straight line, etc.) that adequately describes the experimental drug release profile can be used for either the all-F-wafer or all-S-wafer assemblies. In the present example, the drug release profile observed with the all-S-wafer assemblies can be adequately described, numerically speaking, by a simple zero-order expression, that is:

(2) $$F_{D}^{S}=\;k_{0}\;t$$

where $F_{D}^{S}$ is the percentage of drug released at time *t* and *k*_0_ is the zero-order kinetic constant obtained from the curve fitting. Table 1 summarizes the results from the curve fitting of the release profiles obtained from the all-F-wafer and all-S-wafer stacks.

The results in Table 1 show that a first- and zero-order fit can adequately describe the release profiles of all-F-wafer and all-S-wafer modules, respectively. In each case, the release models explain more than 90% of the total change, as indicated by the *R*^2^ values.

As drug loading (*D_L_*) increases, the rate constant in each case, *k*_1_ and *k*_0_, decreases. Such an effect is attributable to the reduced surface area to volume (SA/V) ratio of the whole assembly, as reported in the literature [7].

Simple linear regression established empirical relationships between *D_L_* and *k*_1_ and *k*_0_, as follows:

(3) $$k_{1}=-0.0008\cdot D_{L}^{F}+0.0567\;R^{2}=0.9845,$$

and

(4) $$k_{0}=-0.0019\cdot D_{L}^{S}+0.2573\;R^{2}=0.9984$$

where the superscripts *F* and *S* denote all-F-wafer and all-S-wafer assemblies, respectively.

As noted above, the release kinetics of the two assembly sections are regarded as being simultaneous and independent. Accordingly, the release kinetics of the whole assembly can be described by a linear combination of the contributions from each assembly section (Equations (1) and (2)), weighed by the respective fractional mass in the assembly, expressed as follows:

(5) $$A_{t}=(p\cdot D_{0})\;[1-\exp(-k_{1}\;t)]+(q\cdot D_{0})\;[0.01\cdot k_{0}\;t]$$

where *A_t_* is the amount of drug released from the assembly at time *t*, *D*_0_ = *D*^F^_L_ + *D*^S^_L_ is the total drug loading, i.e., in the complete assembly (including all-F-wafer and all-S-wafer sections), *p* is the fraction of the total drug loading contained in the all-F-wafer section [F/F/F…/F]*_X_*, and *q* is the fraction of the total dose from the all-S-wafer section [S/S/S…/S]*_Y_*. The rate constants in Equation (5) can be estimated from Equations (3) and (4). For example, for a 30 mg dosage form assembly with a 25:75 F:S ratio, the linear combination expression has the form

(6) $$A_{t}=(0.25\cdot 30)\;[1-\exp(-0.05\cdot t)]+(0.75\cdot 30)\;[0.01\cdot 0.216\cdot \;t]$$

**Figure 6:**
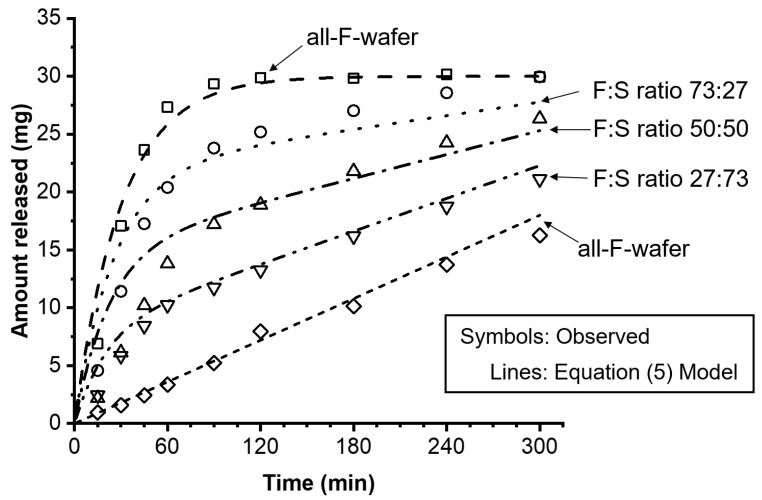
Effect of proportion of all-F-wafer and all-S-wafer sections in assembled dosage form on the drug release of the final assembly. Assemblies were prepared according to the D1 configuration shown in Figure 1. Symbols represent the experimentally observed release profiles. The different lines represent the modeled release profiles using Equations (1)–(5).

Equations (1)–(5) were used to model the experimentally observed drug release profiles of the dosage forms containing 30 mg PGR loading with various F:S wafer ratios. Figure 6 shows the experimental results, represented by open symbols, alongside the release profiles predicted by Equation (5), represented by the various lines.

The drug release kinetics of a modular dosage form can be modeled using a single expression that couples a zero- and a first-order release model (Equation (5)). This expression reasonably describes the level and shape of the release profiles of the modular dosage forms assembled with different F:S wafer ratios. The release kinetic model was quantitatively evaluated using the average fold error (AFE) and absolute average fold error (AAFE) [26]. Table 2 summarizes the drug loading, the fraction of the total drug amount contained in the all-F-wafer and all-S-wafer sections (*p* and *q*, respectively) of the assembly, the zero- and first-order kinetic constants, and the AFE and AAFE values calculated for the modeled release profiles.

The AFE indicates whether the predicted values underestimate or overestimate the observed values. The AAFE shows the absolute error from the actual value. The AFE and AAFE were calculated using the following formulas:

(7) $$AFE=10^{\frac{1}{n}\times \sum \log\left(\frac{A_{t,pred}}{A_{t,obs}}\right)}$$

(8) $$AAFE=10^{\frac{1}{n}\times \sum \left|\log\left(\frac{A_{t,pred}}{A_{t,obs}}\right)\right|}$$

where the subscripts *pred* and *obs* denote predicted (by the model) and observed values, respectively.

A prediction is considered satisfactory if the AAFE value is less than 1.25, acceptable if the AAFE value falls between 1.25 and 2, and poor if the AAFE is greater than 2. Alternatively, the prediction is considered satisfactory if the AFE value falls between 0.8 and 1.25, acceptable if the AFE falls either within the range [0.5–0.8] or [1.25–2], and poor if the AFE value falls either within the range [0–0.5] or is greater than 2 [26].

The overall AFE and AAFE values for the dissolution rate predictions were lower than 1.25, indicating a high level of accuracy and excellent predictive power of the model (Table 2). There was one outlier, F:S 50:50, for which the overall AFE and AAFE were above 1.25 but well below 2. Although the predictions for this particular F:S ratio showed some discrepancies, particularly in overpredicting extent of dissolution at early time points, the model was able to accurately match the dissolution shape and level for the remaining portion of the release profile. Hence, despite the slight deviations, the overall performance of the model for this outlier can still be considered acceptable.

The modeling results indicate that by combining a zero-order expression with a first-order equation, it is possible to obtain reliable estimations of drug release profiles across different F:S ratios. This modeling approach is based on experimental determinations involving only two scenarios: an all-F-wafer and an all-S-wafer. This characteristic of modular dosage forms proves advantageous as it reduces the need for the numerous wet laboratory experiments typically required during the development of traditional solid oral dosage forms.

## 5. Discussion

**Figure 7:**
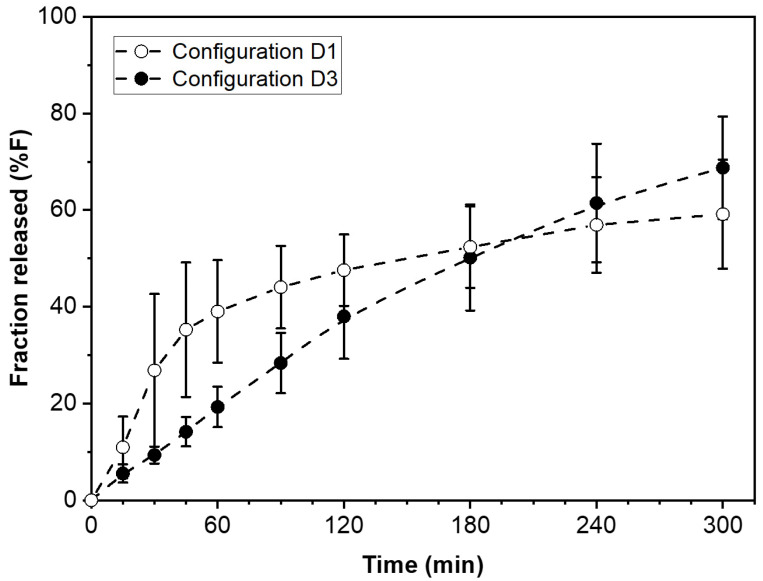
Effect of modular dosage form configuration on drug release kinetics. Comparison of two wafer arrangement configurations, D1 and D3 (see Figure 1) on drug release profile. The two types of modular dosage forms have the same amount of drug, same number of F and S wafers, same and F:S ratio, only different combinatorial configuration of wafers.

The drug release kinetics customization and dose adjustment of the type of modular solid dosage form utilized in this study can both be accomplished using no more than two types of pharmaceutical films to make the necessary wafers. The total number of wafers and the proportion of each type determine the dose and release rate, respectively. The example presented here lumps together all wafers of the same type (D1 configuration in Figure 1). This arrangement resembles a traditional bi-layer tablet. However, the number of possible arrangements for the wafers can indeed be quite large. For a fixed number and proportion of F- and S-wafers, different combinatorial configurations, for example, those depicted in D2 and D3 in Figure 1, provide the ability to further fine-tune the drug release profile. This report focuses on configuration D1, the simplest case of modular dosage form in our approach. Further investigation on the drug release attributes of different wafer configurations used to make the dosage form is warranted. Such a study is out of the scope of the present report. However, Figure 7 presents some preliminary data on the effect of wafer configuration arrangement on drug release, while everything else is kept equal. The graph shows the drug release profiles obtained from modular dosage forms containing the same amount (50 mg) of PGR, and equal proportions of F and S wafers (50:50 F:S ratio). The difference between the two dosage forms is that one has the D1 configuration design, while the other is assembled using configuration D3 (see Figure 1). The D1 configuration results in faster initial drug release. After a cross-over around 180 min, the D3 configuration exhibits greater extent of drug release in the long term. The results in Figure 7 illustrate the degree of flexibility for further adjusting drug release kinetics attainable with the additive manufacturing, multi-layer approach of this study. Such attributes are well suited for controlled release applications.

One crucial point is that the release profiles obtained from the multi-layer stacks do not correspond to the “sum” of the drug release contributions from the individual wafers. Stack assemblies behave as single bodies in terms of their drug release properties, making each arrangement configuration the counterpart of a somewhat different (traditional) formulation [7].

The unique advantages of pharmaceutical films have firmly established them as an important element in the dosage form repertoire of pharmaceutical scientists [8,27,28,29]. The benefits of films in terms of the range of performance, robustness of preparation methods, and suitability for high-resolution analytical methods for quality assessment [30,31] are important elements for their success as dosage forms. One shortcoming of films has been that as a final dosage form, the amount of drug they can carry, and hence deliver, is somewhat limited [13]. However, such limitation arises only as long as the use of films is restricted to their administration into the buccal cavity, which need not be the case. Advances in the development of pharmaceutical films have led to uses not restricted to the buccal cavity [32], and novel film manufacturing methods [23,33] present great potential for expanded uses of films. Application of other methods for prolonging drug release from films [23,24] will further expand their pharmaceutical usefulness. Moreover, multi-layer films are bound to expand the applications of pharmaceutical films further [32,34,35,36]. The use of multiple film layers makes it possible to utilize films to deliver drug amounts similar to those contained in traditional tablets and capsules [7].

Modular dosage forms are a subject of increasing interest, particularly in recent times [37,38,39]. Modularization offers practicable solutions to frequently encountered therapy challenges. Patients often take multiple drugs in separate dosage forms. The so-called “polypill” can be readily implemented utilizing a modular design of dosage forms. An additional advantage is that in the case of chemical incompatibility between drugs, the modular polypill can be configured to keep incompatible drugs physically separate from each other in the dosage form. Moreover, the release of incompatible drugs from the modular dosage form can conceivably be staggered by exploiting the different drug release attributes from different types of wafers in the 3D assembly. 

Precise control of dose, drug release kinetics, and physical location of different drugs in the same dosage form are essential to the much sought-after individualization of drug therapy. The extensive possibilities for reconfiguration of dosage forms afforded by modular design make such dosage forms ideally suited for personalization/individualization of therapies. In considering the advantages of modular dosage forms, one unavoidable and critical aspect is that of cost effectiveness. It is important to point out that the modularization of dosage forms makes it possible to depart from traditional “re-formulation” in favor of “re-configuration” for obtaining a pharmaceutical product with specific performance attributes. Product reconfiguration is necessarily more cost effective because it is based on utilizing prefabricated modules to generate a wide range of product variants. Reconfiguration can be accomplished with a small number of units of the specific variant before committing large amounts of the materials involved. Traditional reformulation, on the other hand, involves committing materials to make a whole batch, which will go to waste if the resulting product does not meet the specified performance requirements.

The manufacture of modular dosage forms relies on the same materials and processes as traditional dosage forms. The difference is not in the type of material or process, but in the manner in which they are utilized. In this study, the functional modules are based on pharmaceutical films. However, modules based on hot-melt extrusion, 3D printing, polymer cups, etc. have been shown to serve as functional modules [37,38,39]. The versatility offered by the modular concept makes it possible to utilize the same functional modules to produce industrially manufactured medicaments or to be utilized in the hospital to assemble dosage forms for the individual patient.

## 6. Conclusions

In this report, the use of films in multiple layers is utilized, and a modular design approach for oral dosage forms of a poorly soluble drug has been presented. The dosage forms consisted of prefabricated modules assembled in a 3D stacking fashion, into the final dosage form. This study shows the advantages of combining the drug release properties of films that exhibit fast and slow drug release. Combining two types of films is presented in its simplest form here, which nonetheless demonstrates the great versatility and utility of the approach. In this study, from an inventory of only two module types, namely, “fast” and “slow” drug release, the drug release rate of the final multi-layer dosage form, for any given drug loading, can be readily adjusted by means of adjusting the proportions of module types. This facile controlled-release method lends itself to adjusting the spring and parachute parameters for enhancing/optimizing the bioavailability of poorly soluble drugs, as well as for the development of patient-centric formulations.

## Figures and Tables

**Table 1:** Summary of the kinetic constants from assemblies of all-S-wafer or all-F-wafer types.

Drug Loading	All-F-Wafer		All-S-Wafer	
	Parameter	Value	Parameter	Value
15 mg	*k*_1_	0.046 ± 0.008	*k*_0_	0.229 ± 0.59
	*R* ^2^	0.9246	*R* ^2^	0.9480
30 mg	*k*_1_	0.032 ± 0.008	*k*_0_	0.198 ± 0.014
	*R* ^2^	0.9329	*R* ^2^	0.9755
50 mg	*k*_1_	0.023 ± 0.002	*k*_0_	0.171 ± 0.018
	*R* ^2^	0.9346	*R* ^2^	0.9920

**Table 2:** Summary of dosage form assembly characteristics and release kinetic modeling.

*D*_0_ (mg)	*q* (S-Section)	*p* (F-Section)	*k*_0_ (%·min^−1^)	*k*_1_ (min^−1^)	AFE	AAFE
30	0.00	1.00	N/A	0.033	1.06	1.09
30	0.27	0.73	0.242	0.039	1.11	1.18
30	0.50	0.50	0.229	0.045	1.31	1.34
30	0.73	0.27	0.216	0.050	1.16	1.16
30	1.00	0.00	0.200	N/A	1.04	1.08

## Data Availability

Not applicable.
